# Using molecular diet analysis to inform invasive species management: A case study of introduced rats consuming endemic New Zealand frogs

**DOI:** 10.1002/ece3.4903

**Published:** 2019-04-13

**Authors:** Bastian Egeter, Cailín Roe, Sara Peixoto, Pamela Puppo, Luke J. Easton, Joana Pinto, Phillip J. Bishop, Bruce C. Robertson

**Affiliations:** ^1^ CIBIO‐InBio, Centro de Investigação em Biodiversidade e Recursos Genéticos Universidade do Porto Vairão Portugal; ^2^ Department of Zoology University of Otago Dunedin New Zealand; ^3^ Faculdade de Ciências da Universidade do Porto Porto Portugal

**Keywords:** diet, *Leiopelma*, predation, primer, rat, trophic

## Abstract

The decline of amphibians has been of international concern for more than two decades, and the global spread of introduced fauna is a major factor in this decline. Conservation management decisions to implement control of introduced fauna are often based on diet studies. One of the most common metrics to report in diet studies is Frequency of Occurrence (FO), but this can be difficult to interpret, as it does not include a temporal perspective. Here, we examine the potential for FO data derived from molecular diet analysis to inform invasive species management, using invasive ship rats (*Rattus rattus*) and endemic frogs (*Leiopelma *spp.) in New Zealand as a case study. Only two endemic frog species persist on the mainland. One of these, *Leiopelma archeyi*, is Critically Endangered (IUCN 2017) and ranked as the world's most evolutionarily distinct and globally endangered amphibian (EDGE, 2018). Ship rat stomach contents were collected by kill‐trapping and subjected to three methods of diet analysis (one morphological and two DNA‐based). A new primer pair was developed targeting all anuran species that exhibits good coverage, high taxonomic resolution, and reasonable specificity. Incorporating a temporal parameter allowed us to calculate the minimum number of ingestion events per rat per night, providing a more intuitive metric than the more commonly reported FO. We are not aware of other DNA‐based diet studies that have incorporated a temporal parameter into FO data. The usefulness of such a metric will depend on the study system, in particular the feeding ecology of the predator. Ship rats are consuming both species of native frogs present on mainland New Zealand, and this study provides the first detections of remains of these species in mammalian stomach contents.

## 1 INTRODUCTION

Although the decline of amphibians has been of international concern for more than two decades, the mechanisms of these declines are often difficult to identify, or they are difficult to disentangle as they may be acting synergistically (Alford, Dixon, & Pechmann, 2001; Alford & Richards, 1999; Stuart et al., 2004). This is exacerbated by a number of amphibian traits that can make them difficult to study, such as spending large portions of time in refugia inaccessible to researchers (e.g., under benthic mud, under deep rock piles), emerging from refugia only ephemerally (on both seasonal and daily timescales), and often being nocturnal. These and other factors have led to an alarming number of species (23%) being placed in the IUCN's Data Deficient category, which is much higher than for the other comprehensively studied vertebrate groups, birds, and mammals (Bishop et al., 2012).

Invasive species are considered one of the most important threats to global biological diversity (Vitousek, Dantonio, Loope, & Westbrooks, 1996; Park, 2004) and are ranked as the third most important detrimental factor affecting amphibian populations (after habitat modification and pollution; Chanson, Hoffman, Cox, & Stuart, 2008). Conservation managers are often tasked with delegating the allocation of resources to the control of invasive species, yet modeling the effects of invasive species on native species can be complex (Lohr et al., 2017). Decisions to implement such control measures are often based on diet studies (Allen & Leung, 2012; Park, 2004). Using morphological methods, successful identification of prey depends on an array of factors including: prey size; the durability of identifiable parts (Major, 1990); the level of digestion prey has been subjected to prior to examination (Veron, 1969); the part of the prey ingested (Day, 1966); and the degree of mastication by the predator (Hansson, 1970; Kasper, Reeson, Cooper, Perry, & Austin, 2004). For example, ship rats (*Rattus rattus*) have often been implicated in the decline of native vertebrate fauna worldwide (Towns, Atkinson, & Daugherty, 2006), but the level of mastication effected by this group makes prey identification from rodent stomach contents notoriously difficult (Hansson, 1970).

Molecular diet analysis can provide the additional tools required to detect prey in predator gastrointestinal or fecal samples, and the diets of a number of rodent species have been investigated using DNA (Lopes et al., 2015; Soininen et al., 2009; Zarzoso‐Lacoste, Corse, & Vidal, 2013). However, there are many considerations to be taken into account when applying DNA‐based diet approaches, such as primer choice, target region, the occurrence of false positives or false negatives, and assay sensitivity (King, Read, Traugott, & Symondson, 2008; Pompanon et al., 2012; Symondson, 2002). A particular challenge is that the abundance of predator DNA can mask prey DNA detections (Vestheim & Jarman, 2008). To overcome this, species‐ or group‐specific primers are often used, targeting the prey of interest, rather than employing broad‐range primers that are likely to co‐amplify DNA from the predator species. Nevertheless, even if predator DNA is not co‐amplified, the relatively high concentration of nontarget DNA can still affect assay sensitivity (Juen, Hogendoorn, Ma, Schmidt, & Keller, 2012; Nejstgaard et al., 2008).

The focus of many diet studies is the contribution of prey species to a given predator species in terms of survival, distribution, energetics, and other aspects of ecological relevance. In such studies, the occurrence and documentation of rare prey species is justifiably considered as being of minor importance. However, when the focus is on determining the impacts of a predator species on prey species of high conservation value, rare occurrences of the prey species can still have major implications for prey populations, especially when the predator density is high, as is often the scenario where invasive species are concerned (Pitt & Witmer, 2007; Pintor, Sih, & Kerby, 2009). Thus, the relative contribution of a prey species to an invasive predator species' diet does not necessarily provide sufficient information to make conservation management decisions (Allen & Leung, 2012). Or worse, it has the potential to mislead conservation practitioners into considering the threat of an invasive species as being minor, due to the high‐value prey species in question occurring at low frequency in the diet.

One of the most common metrics to report in diet studies (both morphological and molecular) is Frequency of Occurrence (FO), the number of diet samples in which a prey species is detected, divided by the total number of diet samples analyzed (Hansson, 1970). Although caution is often advised when interpreting FO data, it has been used for describing dietary composition (Baker, Buckland, & Sheaves, 2014), for ranking the relative importance of various prey to a single predator species (Sinclair & Zeppelin, 2002), for comparing seasonal and regional diet variation of a predator species (Sinclair & Zeppelin, 2002), and for comparing diets among predator species (Murphy, Keedwell, Brown, & Westbrooke, 2005). However, unless additional parameters are incorporated, FO can only ever be a relative measure and cannot be used to estimate the potential impact of a predator species on the prey population (Greenstone, 1996; Szendrei, Greenstone, Payton, & Weber, 2010). This is because FO does not take into account time, an important parameter for determining predation rates (Dempster, 1960; Jones & Toft, 2006).

Here, we examine the potential of FO data derived from molecular diet analysis to inform invasive species management, using invasive ship rats and endemic frogs in New Zealand as a case study.

New Zealand's fauna evolved in the absence of mammals (excluding marine mammals and bats; see Clout & Saunders, 1995), and there are now 31 introduced mammalian species present as wild or feral populations (King, 2005; Parkes & Murphy, 2003), 11 of which are known to consume vertebrates, including hedgehogs (*Erinaceus europaeus*), possums (*Trichosurus vulpecula*), mice (*Mus musculus*), cats (*Felis catus*), pigs (*Sus scrofa*), three rat (*Rattus*) species, and three mustelids (*Mustela*). The New Zealand Government spends over NZD $70 million per year on the control of invasive species (Department of Conservation, 2016) and relies on ecological research to allocate funding to target certain species or geographical areas.

Only four species of native frog remain in New Zealand (all endemic) and only two of those are found on the mainland, in highly fragmented remnant populations; Archey's frog (*Leiopelma archeyi*) and Hochstetter's frog (*Leiopelma hochstetteri*), which are listed as Critically Endangered and Least Concern, respectively (IUCN, 2017). Archey's frog is ranked as the world's most evolutionarily distinct and globally endangered amphibian (EDGE, 2018). *Leiopelma *is an ancient group that has retained unique and evolutionarily basal characteristics not found in most other anuran species (Moffat, 1974; Stephenson, 1952; Worthy, 1987a) and is of high conservation value (Bell, 2010). Archey's frog is an entirely terrestrial species (Bell, Daugherty, & Hitchmough, 1998; Daugherty, Maxson, & Bell, 1982) with intracapsular development, rather than free‐swimming tadpoles (Bell & Wassersug, 2003; Stephenson, 1961). Populations of Archey's frog have declined dramatically in recent years and, although persisting in two regions of New Zealand (Whareorino Forest and Coromadel Peninsula), have not shown signs of recovery (Bell, Carver, Mitchell, & Pledger, 2004; Burns et al., 2018). Hochstetter's frog is semi‐aquatic, usually restricted to streams and seepages in woodland habitats (Crossland, Mackenzie, & Holzapfel, 2005; Green & Tessier, 1990; Nájera‐Hillman, Alfaro, O'Shea et al., 2009; Tessier, Slaven, & Green, 1991), and has a nonfeeding tadpole stage (Bell & Wassersug, 2003; Stephenson, 1955). This species has the most widespread distribution of the *Leiopelma *species, being found in scattered populations over an extensive area of the North Island (Bishop et al., 2013). New Zealand also has three species of introduced frogs (*Litoria *& *Ranoidea*), two of which are declining in their native ranges in Australia and are listed as “Endangered” or “Vulnerable” (IUCN, 2017).

The primary threats to *Leiopelma *are considered to be introduced mammalian predators, infectious disease (chytridiomycosis), and habitat modification (Bishop et al., 2013), but agents of decline have not been conclusively demonstrated (Bishop et al., 2013; Newman et al., 2010). Although it seems, from sporadic reports, that ship rats may represent the greatest mammalian predation threat to New Zealand's frogs (Egeter, Robertson, & Bishop, 2015), the current impacts of introduced predators on New Zealand frog populations are largely unknown (Baber, Moulton, Smuts‐Kennedy, Gemmell, & Crossland, 2006; Bishop et al., 2013; Haigh, Pledger, & Holzapfel, 2007; Tocher & Pledger, 2005). The evidence to date is largely circumstantial: The extinction of three native frog species occurred synchronously with the arrival of introduced fauna (in association with human settlers), as did the range contraction of the currently extant species (Bell, 1994b; Easton et al., 2018; Towns & Daugherty, 1994; Worthy, 1987b).

Indirect predation studies have been carried out comparing *Leiopelma *abundance in areas where mammalian predators had been removed with areas where no predator control had been implemented (reviewed by Egeter, Robertson et al., 2015). The results to date have varied widely in terms of estimating the effects of mammalian predators on *Leiopelma *abundance (see discussion section herein). A major difficulty with comparing frog abundance estimates is that a difference in abundances may not reflect a difference in population size, but only in detection probability, which can vary both spatiotemporally and by observer (Buckland, Goudie, & Borchers, 2000; Crossland et al., 2005; Nájera‐Hillman, King, Alfaro, & Breen, 2009; Yoccoz, Nichols, & Boulinier, 2001). For instance, McLennan (1985) calculated a fourfold difference in abundances of Hochstetter's frogs based on results collected from different observers. *Leiopelma* are also long‐lived (three generations are estimated at 30–45 years for Archey's frog) and produce few eggs (1–22; Bell, 1985; Bell, 1994a; Bell & Wassersug, 2003), so population monitoring necessitates very long‐term studies. Even so, invasive species are more likely to be generalist predators (Dukes & Mooney, 1999), and as such tend to be buffered from fluctuations in the abundance of any one prey species (Inayat et al., 2011). Thus, native amphibian prey populations would not necessarily be expected to fluctuate in tandem with introduced generalist mammals. Diet analysis has the potential to provide estimates of the impact of invasive predators on prey species, as it does not necessarily require long‐term studies, and is not affected by observer bias or frog detection probability across habitats.

To examine the potential of FO data derived from molecular diet analysis to inform invasive species management, we addressed the following objectives: (Bell, 1985, 1994a; Newman et al., 2010) design and validate PCR primers for detecting frog DNA (in terms of specificity, sensitivity, and taxonomic coverage); compare morphological and molecular diet analyses for detecting frog remains in field‐collected ship rat stomach contents; and assess whether the incorporation of a temporal parameter into FO data can provide more informative metrics for making conservation management decisions.

## 2 MATERIALS AND METHODS

### 2.1 Field study

Four sites were visited within two study areas: Whareorino Forest and the Waitakere Ranges. Whareorino Forest is an extensive area of unlogged podocarp‐hardwood forest (Pryde, Lettink, & O'Donnell, 2006) situated in King Country, central North Island, New Zealand, and is managed by local DOC authorities. This area is inhabited by both Hochstetter's and Archey's frogs (Thurley, 1996; Thurley & Bell, 1994). The Waitakere Ranges, Auckland, New Zealand, is largely covered by the Waitakere Ranges Regional Park, administered by the Auckland Regional Council. The Waitakere Ranges are not inhabited by Archey's frogs, but this area was chosen because there are far more distribution data available for Hochstetter's frogs in the Waitakere Ranges than in Whareorino Forest (Allen, 2006; Green, 1994; Green & Tessier, 1990; Moreno, 2009; Nájera‐Hillman, Alfaro, O'Shea et al., 2009; Tessier et al., 1991). Sites in the Waitakere ranges were centered along streams known to be inhabited by Hochstetter's frogs.

At each site, a trapping web consisting of 81 rat snap traps (Victor; Woodstream Corporation) was installed. Each web consisted of 16 trap lines radiating from a central point, each line comprised of five traps, plus an additional trap at the center of the grid. For the initial two trapping sessions, traps had a 25‐m spacing, but the results of these sessions indicated that a low proportion of the rat population present was being trapped. Consequently, for subsequent trapping sessions (*n* = 3) the spacing was decreased to 20 m. Rat traps were baited with peanut butter and placed under wire mesh tunnels with a plastic covering pegged into the ground to reduce the risk to nontarget species. All traps were left baited, but unset for the first night (following Hickson, Moller, & Garrick, 1986; Tobin, Koehler, Sugihara, Ueunten, & Yamaguchi, 1993). Traps were then set for five consecutive nights. Each morning, traps were checked, carcasses removed, and traps reset if necessary. Dissection was carried out at a field station within each study area, whereby whole stomachs (excluding esophagus and intestine) were removed and stored in 95% ethanol. Instruments were washed in ethanol and flamed between dissections.

To ensure that trapping was being carried out on nights that frogs were emerging from diurnal retreats, a 50‐m transect was surveyed each night during trapping. Each survey consisted of visual searches for frogs using torches, as *Leiopelma* rarely produce sounds (Stephenson & Stephenson, 1957). Transects were located near to (within 100 m) trapping grids, but not inside them, to avoid disturbance to trapping. Indices were standardized by always commencing frog counts 1–1.5 hr after dusk (as *Leiopelma *frogs will have left their daytime retreats by this time, given favorable conditions; Cree, 1989) and always completing transects within 30–40 min.

All procedures employed during fieldwork were ethically reviewed and approved by the University of Otago Animal Ethics Committee (ET 25/09).

### 2.2 Primer design and optimization

#### 2.2.1 In silico primer evaluation

Different assays will have different sensitivities for detecting a particular prey species. In order to maximize the detection of frog DNA from stomach content samples, we used two approaches: one using species‐specific primers for each of the two target species, followed by Sanger sequencing; and one using group‐specific primers targeting Anura in general, followed by Illumina MiSeq sequencing.

Species‐specific primer pairs were developed targeting short fragments of the mitochondrial 12S rRNA gene (Table 1), using Primer‐BLAST (http://www.ncbi.nlm.nih.gov/tools/primer-blast) and following the recommendations of King et al. (2008). The 12S mitochondrial DNA region is extensively used as a DNA barcode for identifying vertebrate species (Kocher et al., 1989; Riaz et al., 2011) as it has proved difficult to design primers for the COI region for vertebrates (Deagle, Jarman, Coissac, Pompanon, & Taberlet, 2014). The Primer‐BLAST search included 12S sequences of all five frog species present on mainland New Zealand: Hochstetter's frog (Genbank accession no. DQ28321), Archey's frog (DQ283216), *Ranoidea aurea* (AY819398), *Ranoidea raniformis* (KJ909657), and *Litoria ewingii* (KJ909656).

**Table 1:** Details of final primers designed and used in this study. Underlined sequence represents the Illumina adapter overhang sequences

Target	Primer name	5'−3'	Fragment length (without primers)	Annealing temperature (°C)
*Leiopelma archeyi*	EGETER−2019–12S‐LA‐F	GGCTGGTATCAGGCACATACC	88 bp	69°C
	EGETER−2019–12S‐LA‐R	CCGGCTCTGGTAGCTGTAA		
*Leiopelma hochstetteri*	EGETER−2019–12S‐LH‐F	AACACTAGCCAAGCCGTCGT	84 bp	69°C
	EGETER−2019–12S‐LH‐R	TTCCCTGGCGGAGTGTGAA		
Anura	EGETER−2019–16S‐F	TCGTCGGCAGCGTCAGATGTGTATAAGAGACAGGACCCYATGGARCTTWARAC	150–190 bp	63°C
	EGETER−2019–16S‐R	GTCTCGTGGGCTCGGAGATGTGTATAAGAGACAGTARCTTGGTYCGTTGATCA		

In order to develop an assay to target a broad range of frog species, both for the current study (to detect all four genera of frogs present on mainland New Zealand), and for future studies (in New Zealand or elsewhere), the program AMPLICON (Jarman, 2004) was used to generate primers intended for selectively amplifying anuran DNA from mixed DNA samples. Representative sequences for the 16S rRNA gene from every major anuran superfamily, along with homologous sequences from other species from all animal classes (obtained from NCBI database), were used as input for AMPLICON, with anuran sequences designated as the target group and sequences from all others treated as the excluded group. It should be noted that we initially targeted representative 12S sequences, but no suitable primers were found; hence, 16S sequences were subsequently used.

Resultant primers were tested for specificity and taxonomic coverage in silico using ECOPCR (Ficetola et al., 2010), allowing for up to one mismatched base per primer. Specificity was assessed by testing primers against the entire set of sequences in the NCBI Nucleotide database (downloaded 9th June 2017). To test taxonomic coverage, a separate database, referred to herein as “Anura Database,” was created consisting solely of anuran 16S sequences with one sequence per species and ensuring each sequence contained the primer binding sites. Sequences matching the search term “16S” within the taxon “Anura” in the NCBI Nucleotide database were downloaded (10th June 2017) and mapped to a reference target that included primer binding site sequences in GENEIOUS (v10.1.3; Kearse et al., 2012; see Supporting Information Figure S1 for mapping parameters). Sequences that had less than 100% overlap with the reference sequence were discarded. Using OBITOOLS (v1.2.11; Boyer et al., 2016), one sequence per NCBI Taxonomy ID was extracted to form the final database. This consisted of 4,136 sequences and 3,051 species names that did not contain the terms “sp.”, “cf.,” or “aff.” There are 4,461 anuran species listed in the Nucleotide database not containing the aforementioned terms, so it appears that the Anura Database represents c. 68% of anuran species in the Nucleotide database. The creation of the database ensured that the in silico tests would provide information on the species of frogs that are likely to be missed during PCR, as well as those that are likely to amplify. This is only feasible if all the species in the starting database contain at least one sequence with the anticipated primer binding site, as otherwise ECOPCR can produce false negatives; that is, the absence of an amplified species can be due to the fact that there are simply no target sequences in the original database, rather than being due to primer mismatches. The aim of this step was not to identify every anuran species that might or might not be amplified by the primers; rather it was to identify families within anura that are likely to be underrepresented in mixed DNA samples when using these group‐specific primers. The resulting ECOPCR output was graphed in R (v3.3.2; R Core Team, 2016) using the ROBI suite of packages: ROBITools, ROBIUtils, ROBITaxonomy, and ROBIBarcodes (http://metabarcoding.org/obitools).

#### 2.2.2 In vitro primer evaluation

To assess the specificity of the species‐specific primers, PCRs were performed on DNA from of all five frog species present on mainland New Zealand, as well ship rat DNA. Tissue samples were obtained from the University of Otago (Supporting Information Table S1). DNA was extracted using the DNeasy Blood and Tissue Kit (Qiagen), following the manufacturer's instructions. Gradient PCRs were performed and specificity for the respective species' DNA was confirmed by gel electrophoresis using SYBR Safe (Life Technologies). Final PCR conditions were as follows: 10.5‐µl reactions containing 30–50 ng DNA, 1 × NH_4_ buffer (Bioline), 3.8 mM MgCl_2_ (Bioline), 0.2 mM of each dNTP, 0.5 µM of each primer, and 0.5 U BIOTAQ (Bioline). The thermal cycling profile was an initial step of 94°C for 2 min, then 35 cycles of 94°C for 15 s, 69°C for 25 s, and 72°C for 30 s (Mastercycler Pro 6321, Eppendorf).

To assess the specificity and coverage of the group‐specific anura primers, PCRs were performed on DNA from tissue of 61 frog species from 29 divergent families (Supporting Information Table S1), as well as DNA extracted from tissue of ship rat and a number of other nontarget mammals known to be present in the study sites: Norway rat (*R. norvegicus*), hedgehog (*Erinaceus europaeus*), and human (*Homo sapiens*). Tissue samples were obtained from multiple sources (Supporting Information Table S1). DNA was extracted using the DNeasy Blood and Tissue Kit (Qiagen), following the manufacturer's instructions. Gradient PCRs were performed and specificity for the anuran DNA was confirmed by gel electrophoresis using SYBR Safe (Life Technologies). One primer pair (EGETER‐2019‐16S‐F/R; Table 1) outperformed two others, and final PCR conditions for this pair were as follows: 10‐µl reactions containing 30–50 ng DNA, 5 µl 2X MyTaq HS Mix (Bioline), and 0.5 µM of each primer. Each primer included Illumina adapter overhang sequences, to enable the addition of sample indexes during downstream PCRs (Table 1). The thermal cycling profile was an initial step of 95°C for 10 min; then 35 cycles of 95°C for 30 s, 63°C for 30 s, and 72°C for 30 s; with a final extension of 72°C for 10 min. To assess the taxonomic resolution of sequences generated by this primer pair, PCR products from frog tissue samples were cleaned using Exo/Sap digestion in a final volume of 8ul containing 4 U Exonuclease I (Fermentas) and 1 U Shrimp Alkaline Phosphatase (Fermentas) for 15 min at 37°C and inactivated for 15 min at 85°C, and Sanger‐sequenced in both directions using an ABI 3130xl DNA Analyser (Applied Biosystems).

To test the sensitivity of the assay, we conducted a limit of detection experiment, similar to that performed by Sint, Raso, and Traugott (2012). This experiment consisted of two tests, one using serially diluted total DNA and one using serially diluted amplicon. The concentration of total DNA for two distantly related species (*Ranoidea raniformis *and *Leiopelma hochstetteri*) was measured based on the average of three measurements using the QuBit HS DNA Assay (Thermo Fisher Scientific) and diluted to 2, 0.2, 0.02, 0.002, and 0.0002 ng^–µl^. Separately, PCR product produced by the EGETER‐2019‐16S primers from each of the two species was gel‐extracted using the QIAquick Gel Extraction Kit (Qiagen). The number of amplicon copies in the product was estimated using the QuBit HS DNA Assay (Thermo Fisher Scientific) in conjunction with DNA CALCULATOR (Sint et al., 2012) and dilutions of 1,000, 500, 100, 50, 25, 10, and 1 copy^‐µl^ were made. Five PCR replicates were carried out for each dilution in each test. DNA templates were not mixed prior to PCR: A separate set of replicates was done for each frog species for each test. Furthermore, the entire experiment was carried out twice, once using 1 µl of template for each PCR and once using 1 µl of template plus 1 µl of ship rat total DNA (50 ng^‐µl^).

It should be noted that we also trialed previously published batrachia‐specific primers (Valentini et al., 2016), but were unable to avoid nonspecific amplification of mammalian DNA using the PCR conditions detailed herein (across a gradient of annealing temperatures).

### 2.3 Diet analysis

In the laboratory, morphological analysis of stomach contents was undertaken with the aim of identifying frogs as prey using a dissecting microscope (Olympus SZ61, with Olympus DP25 digital camera attachment, Olympus Corporation) at between 6.7× and 45× magnification. Disposable dissection trays were used for each sample, work surfaces wiped clean with 10% bleach and instruments washed in ethanol and flamed between samples. Reference frog specimens (*R. raniformis*; entire frogs and frog skeletons) were used to compare prey items based on morphological traits commonly used to identify frog remains to order, genus, or species level (Bever, 2005; Holman, 2003; Worthy, 1987a; e.g., the shape of the ilium, femur, radioulna, and tibiofibula, the relative width of digit terminal disks and/or the extent of interdigital webbing; Courtice & Grigg, 1975).

After morphological analysis, samples were homogenized and DNA was extracted following Egeter, Bishop, and Robertson (2015), using the Qiagen DNeasy blood and tissue kit (Qiagen). All PCR plates included two positive controls (DNA from Archey's and Hochstetter's frogs) and negative controls (every eighth well). PCRs were run in duplicate to minimize the impact of stochastic pipetting error and to increase prey detection (Kvitrud, Riemer, Brown, Bellinger, & Banks, 2005; Murphy, Waits, & Kendall, 2003). Pre‐ and post‐PCR procedures were carried out in separate laboratories. Aerosol‐resistant pipette tips were used throughout all PCR procedures. As a qualitative assessment of the prevalence of potential false negatives (e.g., Oehm, Juen, Nagiller, Neuhauser, & Traugott, 2011), where adequate DNA was not extracted or PCR inhibition may have occurred, all stomach samples were subject to PCR using “universal” 12S vertebrate primers (L1091/H1478; Kocher et al., 1989), using PCR conditions as detailed by Egeter, Bishop et al. (2015). This provided an estimation of the number of samples resulting in amplifiable DNA in general. Amplicons from this PCR were visualized on gels but were not sequenced as they are longer (c. 400 bp) than usually recommended for diet analysis studies (King et al., 2008) and would also be expected to amplify ship rat DNA in high proportions.

PCR products from stomach content samples, produced using the species‐specific primer pairs, were cleaned and Sanger‐sequenced as described earlier. PCR products produced using the EGETER‐2019‐16S primer pair were subjected to high‐throughput sequencing. For this, a second PCR was conducted, to add indexes and Illumina flow cell adaptors, using 10‐µl reactions containing: 1 µM of each index‐primer (Gansauge & Meyer, 2013; Kircher, Sawyer, & Meyer, 2012), 5 µl 2X KAPA HiFi HotStart ReadyMix (Kapa Biosystems), and 2 µl of previous PCR product diluted 1:10 using 10 mM Tris. The thermal cycling profile consisted of an initial step of 95°C for 3 min; 10 cycles of 95°C for 30 s, 55°C for 30 s, and 72°C for 30 s; and a final extension of 72°C for 5 min. The resulting PCR product (c. 225–250 bp) was cleaned using 1.2 X by volume AMPure XP beads (Beckman Coulter) following the manufacturer's instructions with the exception that 80% ethanol was used instead of 70%. Elution was done in 25 µl Tris 10 mM. Library quality was assessed by measuring DNA concentration of each cleaned PCR product using Nanodrop 2000 (Thermo Fisher Scientific), and each PCR product was normalized to 15 nM using 10 mM Tris pH 8.5 0.1% Tween. The final pool was created by combining 5 µl from each normalized sample. The quality of the final pool was assessed by qPCR using KAPA Illumina Library Quantification (Illumina) following the manufacturer's instructions. Illumina paired‐end sequencing was performed using a 300‐cycle Illumina MiSeq V2 Kit (Illumina) on an Illumina MiSeq sequencer housed at CIBIO‐InBio (Vairão Campus, Portugal).

### 2.4 Sequence data

Sanger sequences were processed using GENEIOUS (v10.1.3; Kearse et al., 2012). Forward and reverse reads were aligned with 100% similarity and primers were removed. Consensus sequences were visually inspected and, where possible, ambiguities were amended based on chromatograms.

Reads produced on the MiSeq were demultiplexed using BASESPACE (basespace.illumina.com). OBITOOLS (v1.2.11; Boyer et al., 2016) was used for the following: paired‐end reads were aligned, alignments with a score <50 were removed, reads without both primer sequences were removed, reads within each sample were clustered into OTUs only if 100% identical, OTUs <100 bp were removed. OTUs comprising ≤3% of the total read count within a sample were removed. This last threshold was reached by dividing the highest number of reads found in a PCR negative (*n* = 26) by the number of reads in each sample. The highest result for any sample corresponded to 3% of reads. Overall, the filtering resulted in PCR negatives without any remaining reads.

Resultant sequences were BLASTed against the GenBank Nucleotide database using the MEGABLAST (Zhang, Schwartz, Wagner, & Miller, 2000) algorithm. BLAST results were assigned to taxa using MEGAN (Community Edition 6.10.8; Huson et al., 2016) with the default parameters, apart from (minScore = 100.0, topPercent = 5.0, minSupportPercent = 0.0, minSupport = 1). In addition, neighbor‐joining trees were constructed in Mega7 (v7.0.21; Kumar, Stecher, & Tamura, 2016) using a local database, which consisted solely of representative target sequences (160–190 bp) of all five New Zealand mainland frog species, along with sequences derived from stomach samples. A similar tree was constructed for all Sanger‐sequenced frog tissue samples to highlight the efficacy of the target region as a DNA barcode.

### 2.5 Data analysis

Frequency of Occurrence (FO) of frogs as prey for each trapping session was calculated using Equation 1:

(1) $$\text{FO}=\frac{P}{R},$$

where *P* is the number of stomach samples testing positive for frog DNA, and *R* is the number of rats trapped.

We considered that a sample testing positive must represent a minimum of one event when a rat ingested frog tissue; therefore, FO units are presented as minimum number of ingestion events per rat. The minimum number of ingestion events does not necessarily equate to the ingestion or death of one frog as a positive sample may represent multiple ingestion events from more than one frog or, conversely, multiple rats may have consumed the same individual frog, by scavenging portions of a dead frog, for example.

To incorporate a temporal parameter into the FO data, Temporal Frequency of Occurrence (TFO) was also calculated for each 5‐night trapping session in units of minimum number of ingestion events per rat per night (Equation 2).

(2) $$TFO=\left(\sum \frac{P_{i}}{R_{i}}\right)/5,$$

where *i* is the trap night.

Equation 2 assumed that a sample being positive, that is, resulting in sequence(s) matching a frog species, was the result of an ingestion event occurring during the sampling period (i.e., on the night the sample was obtained). We consider that this assumption was likely to hold true due to the following rationale:

- Archey's frogs are active only between dusk and dawn (Cree, 1989), and ship rats are also primarily nocturnal (Dowding & Murphy, 1994; Hooker & Innes, 1995).
- Daylight hours during the study periods ranged from 12 to 15.5 hr.
- Detection probability of frog tissue in ship rat stomach contents using DNA‐based diet analysis under laboratory conditions was previously found to be very low c. 12 hr after ingesting frog tissue (<0.1; Egeter, Bishop et al., 2015). Furthermore, detection probabilities in the laboratory study were likely to be higher than under field conditions as rats in the laboratory were fed ad libitum, which is known to increase detection probabilities over time (Dodd, 2004).
- Therefore, even if a rat ingested frog tissue just before daybreak, but was not caught in a trap until the earliest possible time during the following trapping session (dusk of the same day), frog DNA would not be detected.

This is similar to approaches used by Dempster (1967; see also Ashby, 1974; and Sopp, Sunderland, Fenlon, & Wratten, 1992; Dempster, 1960), but does not assume that the detection of an ingestion event is equivalent to predation of an individual.

## 3 RESULTS

### 3.1 Detecting frog remains in ship rat stomach contents

In total, 191 ship rat stomach content samples were obtained: 60 at Whareorino Forest, where both frog species are present; and 131 in the Waitakere Ranges, where only Hochstetter's frog is present. Of these, 165 (86.4%) exhibited good amplification of vertebrate DNA using the universal vertebrate primers (as indicated by bands in electrophoresis gels). Six of these 165 samples tested positive for the presence of frog remains using molecular diet analysis (Tables 2 and 3; Supporting Information Figure S2).

**Table 2:** Details of trapping sessions and detection of ingestion events including Frequency of occurrence (FO; minimum number of ingestion events per rat) and Temporal Frequency of Occurrence (TFO; minimum number of ingestion events per rat per night)

Study area	Site	Session	Captures	No. of samples positive		FO		TFO	
				LA	LH	LA	LH	LA	LH
Whareorino Forest	1	Mar 2010	16	1	0	0.063	0	0.1	0
Whareorino Forest	2	Mar 2012	44	4	1	0.091	0.023	0.222	0.007
Waitakere Ranges	3	Apr 2010	39	0	0	0	0	0	0
Waitakere Ranges	3	Dec 2011	51	0	1	0	0	0	0.006
Waitakere Ranges	4	Dec 2011	41	0	0	0	0	0	0

Note

LA: Archey's frog; LH: Hochstetter's frog.

**Table 3:** Details of rat stomach samples testing positive for frogs as prey using three diet analysis approaches. All rats were adults

Study area	Site	Sex	Mass (g)	Sample ID	Prey species detected	Detected by species‐specific primer + Sanger	Detected by group primer + MiSeq (final no. of reads)	Detected by morphological analysis
Whareorino Forest	1	F	130	WH15	*L. archeyi*	Y	N	N
Whareorino Forest	2	F	128	WH19	*L. archeyi*	Y	Y (216)	N
Whareorino Forest	2	F	128	WH19	*L. hochstetteri*	N	Y (597)	N
Whareorino Forest	2	F	130	WH45	*L. archeyi*	Y	Y (82)	N
Whareorino Forest	2	M	120	WH35	*L. archeyi*	Y	Y (160)	N
Whareorino Forest	2	F	150	WH17	*L. archeyi*	Y	Y (47)	N
Waitakere Ranges	3	M	130	WA81	*L. hochstetteri*	Y	Y (181)	N

Using morphological analysis, none of the rat stomach contents were found to contain remains of frogs. The species‐specific approach coupled with Sanger sequencing had a similar success rate to the group‐specific approach coupled with MiSeq sequencing (six positives each), but in two cases one of the approaches detected a species the other missed. One sample tested positive for both Hochstetter's frog and Archey's frog (Table 3). In all cases, PCR replicates resulted in the same species being detected. No other species, anuran or otherwise, were detected.

Temporal Frequency of Occurrence (minimum number of ingestion events per rat per night; TFO) ranged from 0 to 0.007 for Hochstetter's frogs and 0.1 to 0.22 for Archey's frogs (Table 2). Only two ingestion events were detected for Hochstetter's frog, one in each study area, despite assaying 191 stomach samples, while four predation events were detected for Archey's frog from 60 stomach samples (contemporary populations of Archey's frogs are not found in the Waitakere Ranges). Ingestion events were detected on nights when 0–0.45 frogs^‐m^ were observed on frog index transects (Supporting Information Table S2). Indeed, on one night that four samples tested positive for frog DNA, no frogs were observed along the transect, indicating that rats were not only predating frogs on nights of high frog emergence (Supporting Information Table S2). No further analyses were conducted on frog emergence data due to the low number of nights with detected ingestion events.

It should be noted that one sample resulted in sequences that were assigned to ship rat, but these were filtered out during the bioinformatic processing. Read numbers from the MiSeq run were lower than expected at only c. 340 reads/sample before filtering and c. 200 final reads/sample (Table 3). This was caused by primer dimers from different primer sets belonging to samples from other unrelated projects that used up a large proportion of the reads in the overall run (data not shown). Nonetheless, as the results are corroborated by the species‐specific primers coupled with Sanger sequencing, this was not deemed to be a major issue.

One drawback of molecular diet analysis is the potential for the occurrence of false positives through sample contamination. We included a PCR negative in every eighth PCR well, as well as using aerosol‐resistant tips and keeping pre‐ and post‐PCR procedures to separate laboratory rooms. The lack of amplification in these sample wells showed that a systematic contamination was not occurring, and contamination of samples without nearby samples also being contaminated would be difficult to explain. Stomach samples resulting in positive detection of frog DNA were not situated close to the positive control on any PCR plates, so this is also unlikely to have caused any false positives.

### 3.2 Group‐specific primers

**Figure 1:**
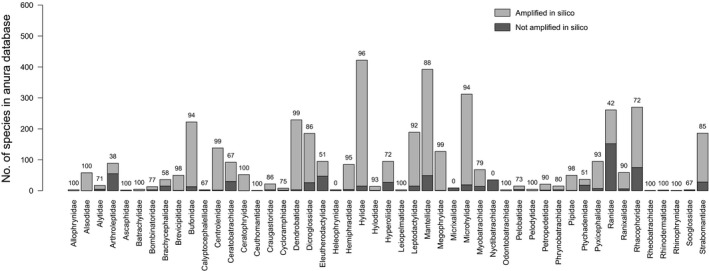
Family coverage of the of EGETER‐2019‐16S primer pair in the order Anura according to in silico PCR using the Anura Database. One base mismatch per primer was allowed. The percentages of each family amplified by the primers are indicated above the bars

In silico, the EGETER‐2019‐16S primers performed well in terms of coverage, amplifying 84% of species in the Anura Database. A few families may be underrepresented by the primer pair, for example, Ranidae (41% species amplified), Arthroleptidae (38%), Eleutherodactylidae (51%), and Ptychadenidae (51%; Figure 1). Amplification and reliable Sanger sequences were obtained from 57/61 (93%) of the species tested in vitro (Supporting Information Table S1). The species not amplified belonged to Ascaphidae, Ranidae, Strabomantidae, and Ptychadenidae, which partially concurs with the in silico analysis where the primers amplified 100%, 42%, 85%, and 51% of these families, respectively.

**Figure 2:**
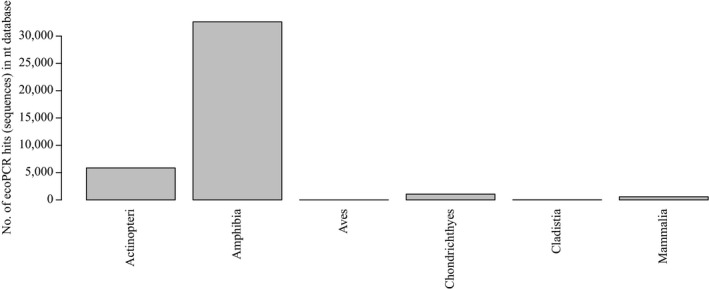
Specificity of EGETER‐2019‐16S primer pair shown as number of entries in the NCBI Nucleotide (nt) database amplified by the primer pair, grouped by Class, according to in silico PCR. One base mismatch per primer was allowed

At lower annealing temperatures (<61°C), DNA from tissue of nontarget (mammalian) species was occasionally amplified, but this did not occur using the final PCR conditions. In silico, 100% of amplifications belonged to the phylum Chordata, 81% of these attributed to the class Amphibia. The remaining amplifications consisted primarily of fish species (Figure 2), indicating there may be some nontarget amplification of this group.

The DNA barcode amplified by EGETER‐2019‐16S primers appears to offer good resolution, unambiguously identifying 83% of the Anura Database to species level and 94% to genus level in silico. Sequences obtained for each species from tissue samples were also unique with a mean p‐distance of 57 base differences (using pairwise deletion of gaps in comparison). The majority of sequences were assigned to the expected taxonomy (Supporting Information Table S1). See Supporting Information Figure S3 for a neighbor‐joining tree highlighting the efficacy of the target region as a DNA barcode.

### 3.3 Limit of detection

**Figure 3:**
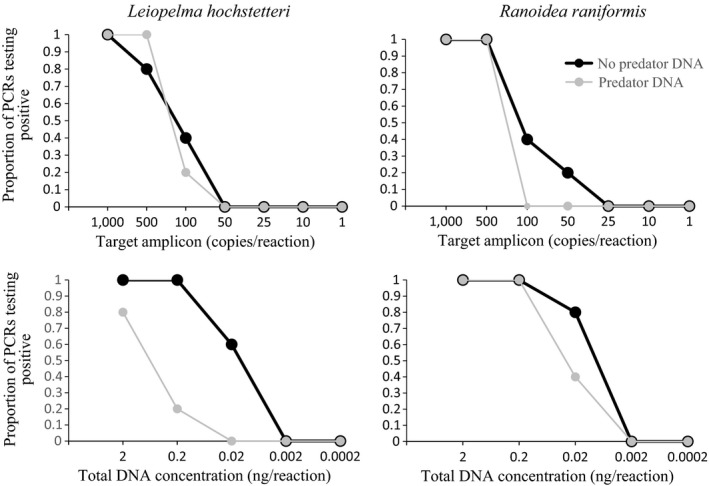
EGETER‐2019‐16S PCR limits of detection for *L. hochstetteri* and *R. raniformis* DNA, using either total DNA (ng/reaction) or target amplicon DNA (copies/reaction), either alone or in the presence of predator (*R. rattus*) total DNA (50 ng). *N* = 5 PCR replicates for each data point

Using the EGETER‐2019‐16S primer pair, DNA could be reliably amplified (100%) in the two frog species tested at 0.2 ng/reaction total DNA (Figure 3). Detection success started to drop at concentrations lower than this, and no amplifications were obtained at 0.002 ng/reaction total DNA. The addition of 50 ng/reaction of predator DNA negatively affected Hochstetter's frog DNA amplification, even at the upper total DNA concentrations, while it only appeared to affect *R. raniformis *DNA amplification at 0.02 ng/reaction (Figure 3). Using target fragment amplicon as template, DNA could be amplified from 500 starting copies (although one PCR out of the five failed at this concentration), regardless of being in the presence of predator DNA or not. At 100 copies/reaction, amplification success was reduced to 0.4 for both frog species and the presence of predator DNA negatively affected amplification at this concentration (Figure 3).

## 4 DISCUSSION

### 4.1 Validation of primers

We present two new species‐specific primers and one group‐specific primer for frogs. The group‐specific primer pair appears to exhibit good coverage, high taxonomic resolution, and reasonable specificity. It was also shown to detect frog DNA at relatively low concentrations, even in the presence of high amounts of predator DNA. However, there were differences in assay sensitivity among the two species tested in the limit of detection experiment, suggesting that variability in the template target DNA or in primer binding sites affects the detection of different prey species, especially when predator DNA is co‐extracted in high relative proportions. Such biases have often been noted using group‐specific primers (see Pinol, Mir, Gomez‐Polo, & Agusti, 2015).

For this study, it was pertinent to ensure that predator DNA was not being amplified, which required relatively high annealing temperatures for all primer sets. If the primers were to be used for other sample types, such as environmental DNA from water bodies, it may be beneficial to test the primers at less stringent conditions to maximize detection of anuran species.

### 4.2 Comparison of diet analysis approaches

From feeding trials, molecular diet analysis has been shown to outperform morphological diet analysis when attempting to detect amphibians as prey in ship rat stomach and fecal samples (Egeter, Bishop et al., 2015), which concurs with the present field‐based study. In fact, studies comparing morphological and molecular diet analyses have generally found that DNA‐based methods improve prey detection success, either by detecting prey more frequently, or by detecting a higher number of prey species (Boyer, Yeates, Wratten, Holyoake, & Cruickshank, 2011; Carreon‐Martinez, Johnson, Ludsin, & Heath, 2011; Casper, Jarman, Gales, & Hindell, 2007; Casper, Jarrnan, Deagle, Gales, & Hindell, 2007; Dunn, Szabo, Mcveagh, & Smith, 2010; Purcell, Mackey, Lahood, Huber, & Park, 2004; Scribner & Bowman, 1998; Soininen et al., 2009; Tollit et al., 2009). In the present study, morphological analysis failed to identify any frog remains in ship rat stomach contents. This is likely because ship rats often do not ingest skeletal components of frog prey, preferring to consume only soft tissue, and even if bones are ingested, they are highly fragmented, making it impossible to discern diagnostic traits (Egeter, Bishop et al., 2015).

The species‐specific and group‐specific molecular diet analysis approaches agreed with each other in five out of the seven detections of frog DNA from ship rat stomach contents. Given the low number of total detections, it is not possible to state whether the disagreements were due to differences in assay sensitivity or PCR stochasticity. Both approaches overcome the issue of co‐amplifying predator or other nontarget DNA, which can hamper prey species detection (see Jarman, Deagle, & Gales, 2004; Zarzoso‐Lacoste et al., 2016; Vestheim & Jarman, 2008). The group‐specific approach reduces the number of PCRs required per sample and has the potential to detect a much broader range of species. Overall, the molecular diet approaches proved to be a valuable addition in this study, allowing the detection of ingestion events that would otherwise have been unobserved.

### 4.3 Incorporation of temporal parameters

FO data provide a metric that can be difficult to interpret, as it does not include a temporal perspective. In this study, we incorporated a temporal parameter into the commonly used FO metric, similar to approaches used by Dempster (1967; see also Ashby, 1974; and Sopp et al., 1992; Dempster, 1960). For each trapping session, this allowed expression in units of minimum number of ingestion events per rat per night (TFO). This unit provides a more intuitive metric, as it constitutes a temporal rate (the minimum number of ingestion events during a given time period), rather than a relative rate (the minimum number of ingestion events per predator). Deagle et al. (2018) noted that when prey are eaten sporadically and in discrete foraging events (as is the case for the present study), FO data may provide meaningful indications of how often a taxon is being consumed. We are not aware of other DNA‐based diet studies that have incorporated a temporal parameter into FO data.

Another benefit of TFO data, as calculated herein, is that the maximum detection period (maximum time that prey is detectable in stomach contents since prey was ingested) is used to ascertain the shortest interval possible between sampling periods. This means that prey DNA detection can be assigned confidently to an ingestion event that occurred within the sampling period, while also maximizing the temporal resolution. Measuring a maximum detection period requires relatively simple feeding trial data, as the aim is only to find the point at which prey are no longer detectable. This is in contrast to measuring 50% detection probabilities from feeding trial data (Gagnon, Doyon, Heimpel, & Brodeur, 2011; Greenstone, Payton, Weber, & Simmons, 2014; Greenstone, Rowley, Weber, Payton, & Hawthorne, 2007; Greenstone et al., 2010; Szendrei et al., 2010; von Berg, Traugott, Symondson, & Scheu, 2008; Waldner, Sint, Juen, & Traugott, 2013; Welch, Schofield, Chapman, & Harwood, 2014), which requires relatively high sample sizes at multiple time points ranging from very high to very low detection probabilities. Furthermore, while 50% detection probabilities can be useful for adjusting relative prey FO data, it is less clear whether it can be reasonably applied to directly adjust a temporal rate, such as TFO, as feeding trial data are unlikely to accurately reflect prey DNA detection across the spectrum of field conditions. This is less of an issue when using a maximum detection period, as the shortest interval possible between sampling periods can be chosen such that detecting prey from a previous sampling period is extremely unlikely.

While it is tempting to assume that each prey detection in molecular diet analysis represents at least one prey individual, this assumption cannot be confirmed for this study as partially eaten Archey's frog carcasses have been found previously with rat bite marks (Fitzgerald & Campbell, 2002; Thurley & Bell, 1994), indicating the possibility of multiple rats consuming tissue from a single frog within one night. Nonetheless, estimating a minimum predation rate (minimum number of individuals consumed during a given time period) from TFO data should be possible in many study systems, particularly those with predators that consume only live whole prey (Codron, Codron, Sponheimer & Clauss 2016; Deagle et al. 2018). If feces are being utilized for diet analysis, rather than stomach contents, then additional considerations are required, but the principles remain the same—an ingestion event can be assigned to a sampling period as long as the fecal sample was produced during the sampling period and the maximum detection period does not extend into the previous sampling period. We recommend that future studies focussed on measuring the impact of predators using molecular diet analyses should take maximum prey detection times into consideration during the design of field sampling, to ensure that each prey detection can be assigned to a specific sampling period.

It should be noted that the estimates we obtained can be considered very conservative. We did not attempt to apply 50% detection probabilities from previous feeding trial data to our field data, which would have adjusted FO values upwards (Gagnon et al., 2011; Greenstone et al., 2014, 2007, 2010; Szendrei et al., 2010) and we assumed that a prey DNA detection was the result of at least one ingestion event, when it may have been the result of numerous events. This means that the true rate of ingestion events is very likely to be higher than the minimum rate estimated herein. Nonetheless, a conservative measure of predation can still provide an informative basis for making conservation management decisions.

A major challenge for molecular diet analysis is to estimate prey biomass or number of prey individuals in a sample. Although relating sequence read counts to prey biomass generally requires significant effort, such as complex feeding trials to calculate correction factors to account for bias between prey taxa, there are promising advances being made in this research area (Bowles, Schulte, Tollit, Deagle, & Trites, 2011; Deagle et al., 2018; Deagle, Thomas, Shaffer, Trites, & Jarman, 2013; Deagle & Tollit, 2007; Thomas, Deagle, Eveson, Harsch, & Trites, 2016; Thomas, Jarman, Haman, Trites, & Deagle, 2014). Another, less commonly applied, approach is to utilize the genetic information of the prey population to estimate the minimum number of prey individuals required to produce the observed variation in a sample (Carreon‐Martinez, Wellband, Johnson, Ludsin, & Heath, 2014). We envisage that the most accurate DNA‐based predation approaches in the future will build on existing methods by combining temporal parameters, FO data, sequence read count data, and individual‐level genetic information.

### 4.4 Predation on New Zealand native frogs

This is the first time that remains of New Zealand native frogs have been detected in mammalian stomach contents. The results indicate that ship rats are consuming both of the mainland species. To make statistical comparisons of the effects of ship rats between the two frog species, more sites and seasons would be required. However, it is notable that detected ingestion events were rare, particularly for Hochstetter's frog—only one detected ingestion event from 133 samples collected from the Waitakere Ranges (of which 112 had amplifiable DNA).

Other studies have compared the abundances of Hochstetter's frogs in areas with or without rodent control, but results to date have been varied (Baber et al., 2008; Egeter, Robertson et al., 2015; Mussett, 2005; Nájera‐Hillman, King et al., 2009). This may be due to difficulties with monitoring Hochstetter's frog abundances as detection probabilities can vary spatially or temporally (Anderson, 2001, 2003; Bailey, Simons, & Pollock, 2004; Crossland et al., 2005; Hyde & Simons, 2001). Nájera‐Hillman, King et al. (2009) found no difference in the relative abundance of Hochstetter's frogs among areas with and without rodent control. Conversely, Mussett (2005) and Baber et al. (2008) found that Hochstetter's frog abundance was higher in mammal‐controlled areas. However, the results of Mussett (2005) were complicated by the fact that the highest ship rat abundance coincided with the highest frog abundance and at some mammal‐controlled sites ship rat abundance was similar to sites without mammal control. Longson, Brejaart, Baber, and Babbitt (2017) observed a fourfold increase in Hochsteter's frog abundances within a mammal‐controlled area over a four‐year period. Using stable isotope analysis, Nájera‐Hillman Alfaro Breen and O'Shea (2009) concluded that shortfin eels (*Anguilla australis*) and banded kokopu (*Galaxias fasciatus*) may be predators of Hochstetter's frogs in the Waitakere Ranges, while the data for ship rats were inconclusive. It is possible that the inconclusive results were due to a very low level of predation by ship rats, which would agree with the results of the present study. Hochstetter's frogs are generally observed in and adjacent to streams and sometimes escape into water when approached (Allen, 2006; Green, 1994; Green & Tessier, 1990; Moreno, 2009; Nájera‐Hillman, Alfaro, O'Shea et al., 2009; Tessier et al., 1991), which may help to explain the low number of ingestion events detected in this study. However, more sampling would be required to ascertain whether ingestion events are indeed a consistently rare event across various sites and seasons.

At Whareorino Forest, five ingestion events of Archey's frog were detected out of 60 samples (of which 54 had amplifiable DNA). Considering the TFO values of 0.1–0.22 ingestion events per rat per night, this equates to 0.2–1.18 ingestion events per ha per night, based on the number of rats caught in the effective trapping area during the sampling periods (and Brown, Moller, Innes, & Alterio, 1996; following Hooker & Innes, 1995). Archey's frog densities can be high, estimated at c. 34 and 77 frogs per 100 m^2^ on two monitoring grids in Whareorino Forest in 2011 (Pledger, 2011). However, as they are long‐lived and produce few eggs (Bell, 1985, 1994a; Bell & Wassersug, 2003), such a frequency of ingestion events may still have a significant impact on the population. With the current data, this remains difficult to interpret and these rates are also likely to change over time, given the annual fluctuation of rat densities (e.g., Daniel 1972; Smith 1986) and varying food sources available. Results of a previous experiment indicated that population sizes of Archey's frogs decreased outside a rodent‐controlled area, while they remained stable or increased inside the rodent‐controlled area, although it should be noted that the study was confined to a small sample size of two 100‐m^2^ monitoring grids per treatment (Pledger, 2011). Sites 1 and 2 of the present study were situated close to the grid from that experiment, and our results provide some support for those findings.

The results of this study were provided to the New Zealand Department of Conservation and this, along with multiple lines of evidence indicating the negative impact of introduced mammals on a range of native species, has led to the inclusion of Whareorino Forest in New Zealand's mammal control program.

## 5 CONCLUSIONS

Ship rats are consuming both species of native New Zealand frogs still present on the mainland. This is the first time these species have been detected in mammalian stomach contents. Molecular diet analysis outperformed morphological techniques. Although frog predation by ship rats was rare, it may still have a significant impact on the frog populations. We were able to incorporate a temporal parameter into FO diet data, which allowed the calculation of ingestion events per rat per night. We are not aware of other DNA‐based diet studies that have incorporated a temporal parameter into FO data. The usefulness of such a metric will depend on the study system, in particular the feeding ecology of the predator. We provide recommendations for future diet studies focussed on measuring the impact of predators on prey species.

## AUTHOR CONTRIBUTIONS

BE designed the study and the primers, carried out the field work, and led the manuscript writing. PJB and BCR guided the research. BE, CR, SP, PP, and JP conducted the laboratory work. LJE contributed to the analysis of the results. All authors provided manuscript input and edits and participated in discussions that developed the work.

## Data Availability

Fasta files containing all Sanger‐sequenced tissue‐derived sequences and final OTUs from stomach‐derived sequences have been submitted to Dryad https://doi.org/10.5061/dryad.0ds81v1.
