# Supplementary material for: Using molecular diet analysis to inform invasive species management: A case study of introduced rats consuming endemic New Zealand frogs
Source: Ecol Evol. 2019 Apr 13;9(9):5032–48. doi: 10.1002/ece3.4903 (PMC6509367; doi:10.1002/ece3.4903)
Supplement: Supplementary file 1 [file ECE3-9-5032-s001.docx]

**Using molecular diet analysis to inform invasive species management: A case study of introduced rats consuming endemic New Zealand frogs**

Bastian Egeter, Bruce C. Robertson, Cailin Roe, Sara Peixoto, Pamela Puppo, Luke J. Easton, Joana Pinto, Phillip J. Bishop


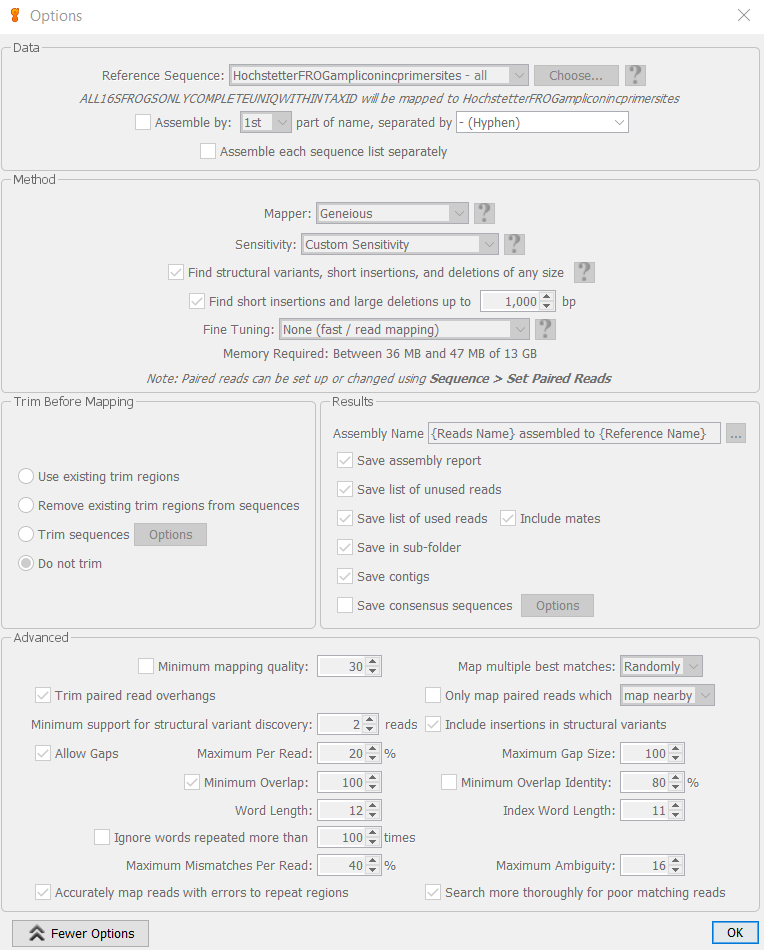


Fig S1 Mapping parameters used in GENEIOUS to map anuran 16S sequences downloaded from Nucleotide database to a reference fragment that included primer binding sites.

Table S1 Species included in in vitro testing of group-specific primer tests. Also indicated are the taxon assignment results following treatment of Sanger sequences using GENEIOUS and MEGAN parameters as detailed in the main text. FM - Field Museum of Natural History (Chicago, Illinois, USA), NMNH - Smithsonian Institution National Museum of Natural History (Washington, DC, USA), TUB - Technical University of Braunschweig (Braunschweig, Germany), AM - Australian Museum (Sydney, Australia), UO - University of Otago (Dunedin, New Zealand), CIBIO - Centro de Investigação em Biodiversidade e Recursos Genéticos (Porto, Portugal).

| **ID** | **Source** | **Source ID** | **Family** | **Genus** | **Species** | **Sequence obtained from Sanger sequencing** | **Assigned taxon** | **Reason for inaccuracy** |
| --- | --- | --- | --- | --- | --- | --- | --- | --- |
| GF76 | CIBIO | GVA1935 | Alytidae | Discoglossus | scovazzi | Y | Discoglossus pictus scovazzi |  |
| GF5 | FM | 272053 | Arthroleptidae | Arthroleptis | stenodactylus | Y | Arthroleptis stenodactylus |  |
| GF33 | TUB | ZCMV8424 | Arthroleptidae | Leptopelis | bocagii | Y | Leptopelis vermiculatus | Fragment not in Nucleotide database for this species |
| GF12 | FM | 282797 | Arthroleptidae | Leptopelis | vermiculatus | Y | Leptopelis vermiculatus |  |
| GF52 | UO | Ascaphus1 | Ascaphidae | Ascaphus | sp. | N |  |  |
| GF48 | TUB | ZCMV12654 | Bombinatoridae | Bombina | orientalis | Y | Bombina orientalis |  |
| GF26 | NMNH | USNM533993 | Brachycephalidae | Ischnocnema | ramagii | Y | Ischnocnema ramagii |  |
| GF15 | FM | 274834 | Brevicipitidae | Breviceps | mossambicus | Y | Breviceps mossambicus |  |
| GF43 | TUB | ZCMV11016 | Bufonidae | Duttaphrynus | melanostictus | Y | Duttaphrynus melanostictus |  |
| GF3 | FM | 231189 | Bufonidae | Pedostibes | hosii | Y | Limnonectes kuhlii | Better match to this species |
| GF69 | CIBIO | 11722 | Bufonidae | Sclerophrys | xeros | Y | Sclerophrys xeros |  |
| GF20 | NMNH | USNM534153 | Centrolenidae | Hyalinobatrachium | pulverata | Y | Hyalinobatrachium pulveratum |  |
| GF18 | NMNH | USNM534194 | Craugastoridae | Craugastor | noblei | Y | Craugastor noblei |  |
| GF17 | NMNH | USNM284536 | Cycloramphidae | Cycloramphus | fuliginosus | Y | Cycloramphus | Many sequence ambiguities |
| GF24 | NMNH | USNM313819 | Dendrobatidae | Dendrobates | auratus | Y | Dendrobates |  |
| GF72 | CIBIO | 7748 | Dicroglossidae | Hoplobatrachus | occipitalis | Y | Hoplobatrachus rugulosus | Better match to this species |
| GF22 | NMNH | AJC0850 | Hemiphractidae | Hemiphractus | fasciatus | Y | Hemiphractus fasciatus |  |
| GF35 | TUB | ZCMV11025 | Hylidae | Agalychnis | callidryas | Y | Agalychnis callidryas |  |
| GF38 | TUB | ZCMV12655 | Hylidae | Dryophytes | cinereus | Y | Dryophytes cinereus |  |
| GF67 | UO | L. aure1 | Hylidae | Litoria | aurea | Y | Litoria aurea |  |
| GF66 | UO | L. ewing1 | Hylidae | Litoria | ewingii | Y | Litoria | L. paraewingi has close score, grouped within 5% threshold |
| GF65 | UO | L. rani1 | Hylidae | Litoria | raniformis | Y | Litoria aurea | Fragment not in Nucleotide database for this species |
| GF30 | NMNH | USNM286958 | Hylidae | Phyllomedusa | trinitatis | Y | Phyllomedusa | Identical match for two species |
| GF4 | FM | 282770 | Hylidae | Pseudacris | triseriata | Y | Pseudacris | Identical match for three species |
| GF16 | NMNH | USNM303070 | Hylidae | Scinax | crospedospilus | Y | Scinax crospedospilus |  |
| GF19 | NMNH | USNM284550 | Hylodidae | Hylodes | sp | Y | Crossodactylus | ? |
| GF42 | TUB | ZCMV8414 | Hyperoliidae | Afrixalus | fornasini | Y | Afrixalus fornasini |  |
| GF34 | TUB | ZCMV12369 | Hyperoliidae | Heterixalus | betsileo | Y | Heterixalus betsileo |  |
| GF37 | TUB | ZCMV8415 | Hyperoliidae | Hyperolius | argus | Y | Hyperolius sp. TNHC 61197 | Fragment not in Nucleotide database for this species |
| GF74 | CIBIO | 4680 | Hyperoliidae | Kassina | senegalensis | Y | Kassina senegalensis |  |
| GF64 | UO | L. archeyi2 | Leiopelmatidae | Leiopelma | archeyi | Y | Leiopelma archeyi |  |
| GF63 | UO | L. hochstetteri1 | Leiopelmatidae | Leiopelma | hochstetteri | Y | Leiopelma hochstetteri |  |
| GF23 | NMNH | USNM287011 | Leptodactylidae | Leptodactylus | fuscus | Y | Leptodactylus fuscus |  |
| GF28 | NMNH | USNM319590 | Leptodactylidae | Physalaemus | biligonigerus | Y | Physalaemus biligonigerus |  |
| GF47 | TUB | ZCMV12379 | Mantellidae | Aglyptodactylus | madagascariensis | Y | Aglyptodactylus madagascariensis |  |
| GF36 | TUB | ZCMV12215 | Mantellidae | Mantidactylus | femoralis | Y | Mantidactylus mocquardi | Better match to this species |
| GF8 | FM | 273702 | Megophryidae | Leptobrachella | mjobergi | Y | Leptobrachella | L. juliandringi has close score, grouped within 5% threshold |
| GF9 | FM | 270739 | Megophryidae | Leptobrachium | smithi | Y | Leptobrachium smithi |  |
| GF11 | FM | 231143 | Megophryidae | Leptolalax | dringi | Y | Leptolalax fritinniens | Fragment not in Nucleotide database for this species |
| GF10 | FM | 272564 | Megophryidae | Oreolalax | omeimontis | Y | Oreolalax | Identical match for two species |
| GF13 | FM | 252956 | Microhylidae | Calluella | guttulata | Y | Calluella guttulata |  |
| GF21 | NMNH | USNM302429 | Microhylidae | Elachistocleis | sp | Y | Elachistocleis | Identical match for three species |
| GF25 | NMNH | USNM523962 | Microhylidae | Glyphoglossus | molossus | Y | Glyphoglossus molossus |  |
| GF14 | FM | 269668 | Microhylidae | Kalophrynus | pleurostigma | Y | Kalophrynus pleurostigma |  |
| GF39 | TUB | ZCMV11017 | Microhylidae | Kaloula | pulchra | Y | Kaloula pulchra |  |
| GF27 | NMNH | USNM-FS36487 | Microhylidae | Microhyla | inornata | Y | Micryletta heymonsi | Better match to this species |
| GF29 | NMNH | USNM523970 | Microhylidae | Microhyla | ornata | Y | Microhyla | M. fissipes has close score, grouped within 5% threshold |
| GF7 | FM | 260137 | Microhylidae | Rhombophryne | alluaudi | Y | Rhombophryne alluaudi |  |
| GF32 | NMNH | USNM336377 | Microhylidae | Scaphiophryne | calcarata | Y | Scaphiophryne calcarata |  |
| GF45 | TUB | ZCMV12374 | Microhylidae | Stumpffia | roseifemoralis | Y | Stumpffia sp. Ca33 | Fragment not in Nucleotide database for this species |
| GF2 | AM | R.148188.001 | Myobatrachidae | Crinia | signifera | Y | Crinia signifera |  |
| GF1 | AM | R.167566.001 | Myobatrachidae | Limnodynastes | tasmaniensis | Y | Limnodynastes tasmaniensis |  |
| GF6 | FM | 259980 | Pelobatidae | Scaphiopus | holbrookii | Y | Scaphiopus holbrookii |  |
| GF70 | CIBIO | 9573 | Phrynobatrachidae | Phrynobatrachus | sp. | Y | Phrynobatrachus francisci | Only match |
| GF41 | UO | X. laevis1 | Pipidae | Xenopus | laevis | Y | Xenopus | Identical match for two species |
| GF44 | TUB | ZCMV8425 | Ptychadenidae | Ptychadena | mascareniensis | N |  |  |
| GF73 | CIBIO | 11815 | Ptychadenidae | Ptychadena | trinodis | Y | Ptychadena | Fragment not in Nucleotide database for this species |
| GF46 | TUB | ZCMV12656 | Ranidae | Hylarana | sp | N |  |  |
| GF40 | TUB | ZCMV11031 | Ranidae | Rana | dalmatina | Y | Rana dalmatina |  |
| GF49 | TUB | ZCMV11015 | Rhacophoridae | Rhacophorus | dennysi | Y | Rhacophorus dennysi |  |
| GF31 | NMNH | USNM268942 | Strabomantidae | Pristimantis | fenestratus | N |  |  |


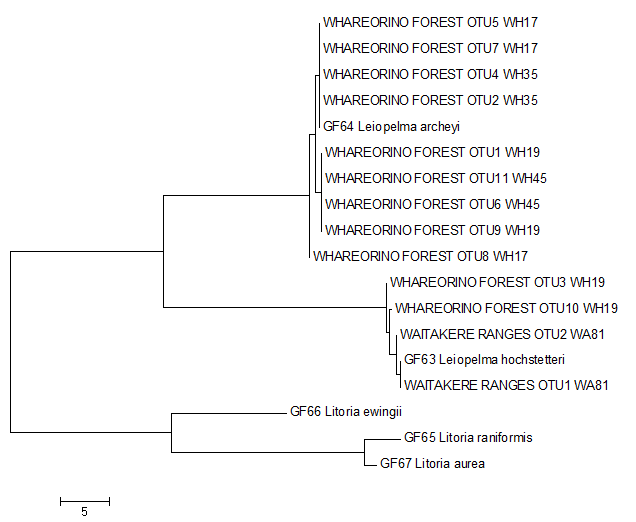


Fig S2 Neighbour joining tree of representative sequences of all New Zealand mainland frog species along with stomach sample-derived sequences. Units are the number of base differences per sequence. Only positions that had base calls for all sequences were included (i.e. positions containing gaps for one or more sequences were removed). There were a total of 194 base positions used to generate the tree.

Table S2 Nightly trapping data for all trapping sessions.

| **Study area** | **Site** | **Session** | **Trapping night** | **Rats caught** | **Positive for *L. archeyi* DNA (n)** | **Positive for *L. hochstetteri* DNA (n)** | **Frogs observed on index transect (frogs/m)** | **Date** |
| --- | --- | --- | --- | --- | --- | --- | --- | --- |
| whareorino | 1 | Mar-10 | 1 | 4 |  |  | 0.02 | 22/03/2010 |
| whareorino | 1 | Mar-10 | 2 | 6 |  |  | 0.086 | 23/03/2010 |
| whareorino | 1 | Mar-10 | 3 | 4 |  |  | 0.086 | 24/03/2010 |
| whareorino | 1 | Mar-10 | 4 | 0 |  |  | 0.076 | 25/03/2010 |
| whareorino | 1 | Mar-10 | 5 | 2 | 1 |  | 0.162 | 26/03/2010 |
| whareorino | 2 | Mar-12 | 1 | 27 | 3 | 1 | 0.00 | 07/03/2012 |
| whareorino | 2 | Mar-12 | 2 | 6 |  |  | 0.00 | 08/03/2012 |
| whareorino | 2 | Mar-12 | 3 | 5 |  |  | 0.00 | 09/03/2012 |
| whareorino | 2 | Mar-12 | 4 | 5 |  |  | 0.12 | 10/03/2012 |
| whareorino | 2 | Mar-12 | 5 | 1 | 1 |  | 0.45 | 11/03/2012 |
| waitakere | 3 | Apr-10 | 1 | 8 |  |  | 0.02 | 05/04/2010 |
| waitakere | 3 | Apr-10 | 2 | 9 |  |  | 0.24 | 06/04/2010 |
| waitakere | 3 | Apr-10 | 3 | 12 |  |  | 0.02 | 07/04/2010 |
| waitakere | 3 | Apr-10 | 4 | 6 |  |  | 0.00 | 08/04/2010 |
| waitakere | 3 | Apr-10 | 5 | 4 |  |  | 0.00 | 09/04/2010 |
| waitakere | 3 | Dec-11 | 1 | 34 |  | 1 | 0.02 | 29/11/2011 |
| waitakere | 3 | Dec-11 | 2 | 8 |  |  | 0.00 | 30/11/2011 |
| waitakere | 3 | Dec-11 | 3 | 6 |  |  | 0.00 | 01/12/2011 |
| waitakere | 3 | Dec-11 | 4 | 3 |  |  | 0.00 | 02/12/2011 |
| waitakere | 3 | Dec-11 | 5 | 0 |  |  | 0.00 | 03/12/2011 |
| waitakere | 4 | Dec-11 | 1 | 13 |  |  | 0.10 | 13/12/2011 |
| waitakere | 4 | Dec-11 | 2 | 14 |  |  | 0.12 | 14/12/2011 |
| waitakere | 4 | Dec-11 | 3 | 8 |  |  | 0.10 | 15/12/2011 |
| waitakere | 4 | Dec-11 | 4 | 2 |  |  | 0.08 | 16/12/2011 |
| waitakere | 4 | Dec-11 | 5 | 4 |  |  | 0.00 | 17/12/2011 |


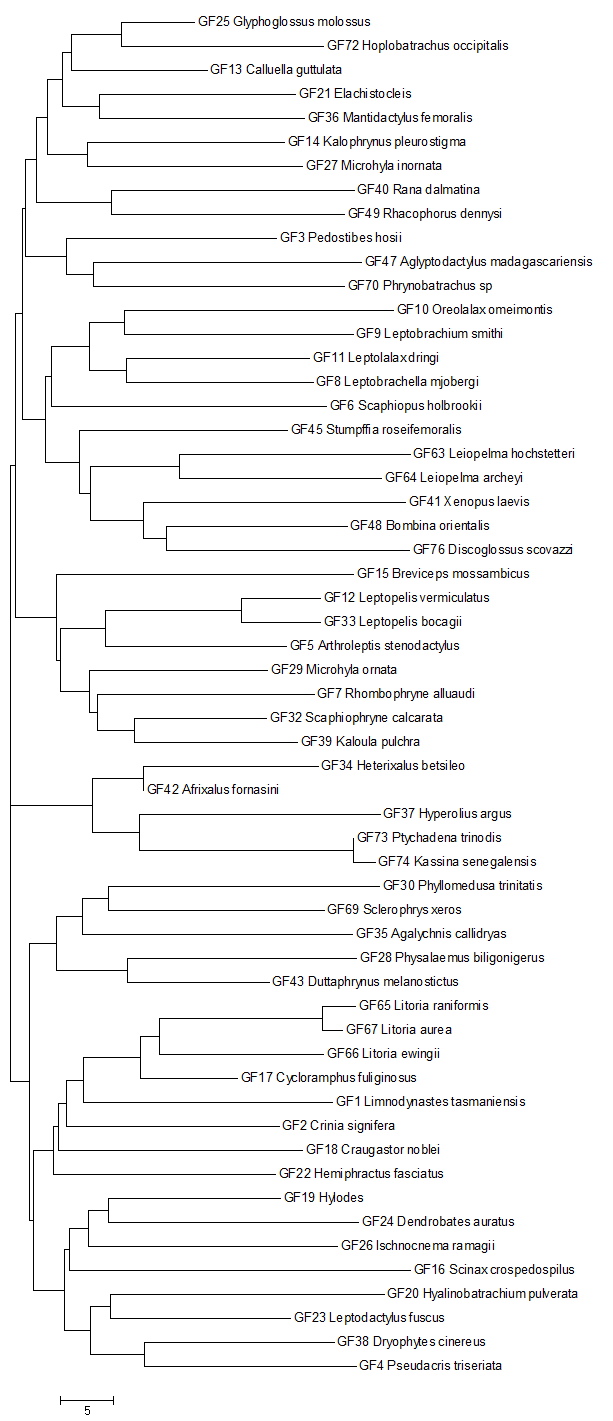


Fig S3 Neighbour joining tree of all sequences obtained using the EGETER-2019-16S primer pair. In the majority of cases each species appears to be well defined, highlighting the efficacy of the target region as a DNA barcode. Units are the number of base differences per sequence. Alignment gaps are included in the analysis. There were a total of 202 base positions used to generate the tree.
